# Occurrence of Ochratoxin A in the Wild Boar (*Sus scrofa*): Chemical and Histological Analysis

**DOI:** 10.3390/toxins4121440

**Published:** 2012-12-04

**Authors:** Giancarlo Bozzo, Edmondo Ceci, Elisabetta Bonerba, Angela Di Pinto, Giuseppina Tantillo, Elvira De Giglio

**Affiliations:** 1 Department of Veterinary Public Health, Faculty of Veterinary Medicine, University of Bari, Valenzano, Bari 70010, Italy; E-Mails: giancarlo.bozzo@uniba.it (G.B.); elisabetta.bonerba@uniba.it (E.B.); angela.dipinto@uniba.it (A.P.); giuseppina.tantillo@uniba.it (G.T.); 2 Department of Chemistry, Faculty of Sciences, Mathematics, Physics and Naturals, University of Bari, Via Orabona, Bari 70125, Italy; E-Mail: elvira.degiglio@uniba.it

**Keywords:** ochratoxin A, *Sus scrofa*, wild boar

## Abstract

Ochratoxins are fungal secondary metabolites that may contaminate a broad variety of foodstuffs, such as grains, vegetables, coffee, dried fruits, beer, wine and meats. Ochratoxins are nephrotoxins, carcinogens, teratogens and immunotoxins in rats and are also likely to be in humans. In 2009/2010, a survey of the presence of Ochratoxin A (OTA) in regularly hunted wild boars in the Calabria region of southern Italy detected OTA in 23 animals in the kidney, urinary bladder, liver and muscles: 1.1 ± 1.15, 0.6 ± 0.58, 0.5 ± 0.54 and 0.3 ± 0.26 μg/kg, respectively. Twelve tissue samples showed levels of OTA higher than the guideline level (1 μg/kg) established by the Italian Ministry of Health. In five wild boars, gross-microscopic lesions were described for the organs displaying the highest concentrations of OTA determined by HPLC-FLD analysis, *i.e*., the kidney, liver and urinary bladder.

## 1. Introduction

Ochratoxins are fungal secondary metabolites that contaminate grains, legumes, coffee, dried fruits, beer and wine, and meats [1]. Ochratoxins are potent nephrotoxins, carcinogens, teratogens and immunotoxins in rats and also likely to be in humans [2,3]. Ochratoxins are produced by members of two fungal genera: the Aspergillus species, *A. ochraceus*, *A. melleus*, *A. auricomus*, *A. ostianus*, *A. petrakii*, *A. sclerotiorum* and *A. sulfureus*, all of which are classified in the section Circumdati (also called the A. ochraceus group); *A. alliaceus* and *A. albertensis*, formerly placed in the section Circumdati and recently reclassified in the section Flavi [4]; *A. niger* and *A. carbonarius* (in the section Nigri); *A. glaucus* (or Eurotium herbariorum, section Aspergillus); and *Penicillium verrucosum* [5,6,7]. The presence of ochratoxins in food has been reported worldwide. Mycotoxic porcine nephropathy (MPN) was comprehensively reviewed by Krogh [8], and ochratoxin A (OTA) is regarded as the possible cause of this nephropathy [9]. OTA is highly nephrotoxic and may cause both acute and chronic kidney lesions. Accordingly, OTA has been suspected to be involved in the aetiology of Balkan endemic nephropathy (BEN), a human disease characterized by progressive renal fibrosis and by tumors of the urinary tract [10,11,12,13], such as carcinoma of the renal pelvis, ureters and bladder [2,14]. Similarly, neoplasia (fibroma, adenoma and fibro-adenoma) in kidneys has been reported in Bulgarian cases of MPN [15].

The climate of southern Europe, with its warm temperatures and high humidity, associated with poor grain storage practices, favors the production of OTA [16,17,18]. OTA is a moderately stable molecule of mw 403.81 (C_20_H_18_ClNO_6_,) (IARC Classification: 2B) that remains unaltered during most food transformation processes. In addition, OTA may undergo bio-concentration in some animal tissues/organs, leading to higher concentrations in meat products [19,20,21].

Regulations on mycotoxins have been established in many countries to protect consumers from the harmful effects of these compounds. Different factors play a role in the decision-making process to set safe limits for mycotoxins. These include scientific factors, for example, the availability of toxicological and epidemiological data, detailed knowledge about possibilities for sampling and analysis, and socioeconomic issues [3,22]. The Commission Regulation EC No 1881/2006 of 19 December [23] does not establish maximum OTA levels in meat-based products. However, the Italian Ministry of Health Circular No 10, dated 9 June 1999 [24], establishes, as a guideline, a value for swine meat and meat products: the OTA maximum level of 1 μg/kg.

In 2009/2010, during a pilot program to survey the presence of OTA in regularly hunted wild boars in the Calabria region of southern Italy, OTA was detected in twenty-three animals in various tissues and organs. OTA levels and effects in wild boars are poorly documented, so the aim of this study was to investigate the presence of OTA in the tissues and organs of these animals. The purpose was to detect the toxin in tissues and organs of the animals in order to describe OTA-related gross and histological lesions and to compare the data with a previous study on OTA in domestic swine [25].

## 2. Experimental Section

### 2.1. Samples

A total of 23 wild boars hunted in the Calabria region were obtained and analyzed to estimate the presence of OTA. Samples of the kidney, urinary bladder, liver and muscles were collected and divided into two portions, one which was immediately frozen and one preserved in 10% neutral buffered formalin.

### 2.2. Analytical Reagents and Calibration

A 10× concentrate of phosphate buffered saline PBS was purchased from Vicam (part #G1113). 10× PBS concentrate should be diluted to 1× with purified water as needed, *i.e*., a dilute of 100 mL of 10× concentrate with 900 mL purified water.

Ochratoxin A (Supelco; product no. 46912), packaged in sealed ampoules at a concentration of 50 ng/µL in benzene: acetic acid (99:1) was used as an analytical standard. This standard was prepared according to the AOAC official methods. The standards for the OchraTest by HPLC were prepared as follows: (a) Ochratoxin-working solution 1—a dilution of 1:50 of ochratoxin A standard (50 ng/µL), 20 μL of ochratoxin A (50 ng/μL) + 980 μL ethyl alcohol; (b) Ochratoxin-working solution 2, prepared by diluting ochratoxin-working solution 1 (1 ng/μL) 10 times, 100 μL of ochratoxin-working solution 1 (1 ng/μL) + 900 μL ethyl alcohol. The calibration curve was obtained by diluting ochratoxin-working solution 2 with methanol in order to obtain the following concentrations: 1.0, 5.0, 10.0, 20.0 and 50.0 (ng/mL). Each solution was injected three times, and mean values were used to construct the five-point calibration curve, *r*-squared 0.9995.

### 2.3. Apparatus and Chromatographic Conditions

For liquid chromatography analysis, an Agilent 1100 Series instrument equipped with pumps, a Rheodyne Model 7125 injector (100 µL loop, full-loop injection) and a fluorescence detector were used. An LC Restek column C18 (5 µm) (250–4.6 mm internal diameter) was used with a mobile phase consisting of a mixture of water: acetonitrile: acetic acid (49.5:49.5:1 by volume), degassed at a flow rate of 0.9 mL/min. Detection of OTA was carried out using 333 and 477 nm as wavelengths for excitation and emission, respectively.

### 2.4. Sample Extraction and Clean-up of Tissues and Organs

A 20 g aliquot of wild boar tissue was homogenized with 6 mL of 1 M phosphoric acid in a Ultra Turrax T25 homogenizer for 2–3 min. A 2.5 g aliquot of the homogenate was transferred into a Pyrex centrifuge tube, extracted twice with 5 mL of ethyl acetate and centrifuged for 5 min at 350 *g*. The organic phases were combined, reduced to approximately 3 mL with a rotary evaporator and back-extracted one time with 3 mL of 0.5 M NaHCO_3_ [26]. The aqueous extract was loaded on to an OchraTest WB column (Vicam), which contained a specific antibody for ochratoxin A. After washing with 10 mL of PBS Buffer and 10 mL of water, the mycotoxin was eluted with 1.5 mL of methanol. The elution is evaporated to dryness and then dissolved with a mobile phase to inject onto the HPLC.

### 2.5. Samples for Histological Testing

The gross lesions were examined and recorded in the laboratory. Samples of the kidney, urinary bladder, liver and muscle were collected for histological examination, with tissue samples being fixed in 10% neutral buffered formalin. The samples were embedded in paraffin wax, and the blocks were sectioned at 4 μm and stained with haematoxylin and eosin (H&E).

## 3. Results

Analytical methods using an immunoaffinity column and HPLC with fluorometric detection for the quantification of ochratoxin A in organs and tissues have been described by various authors [26,27]. The results of recovery experiments of the full analytical procedure implemented herein showed that the overall average recovery (mean of means) in the tested range of concentrations was 96.4 ± 2.9%, obtained with triplicate injections of spiked sample extract at different concentration levels (5, 10 and 50 μg/kg) before the clean-up procedure. The limit of detection (LOD) and the limit of quantitation (LOQ) of the method were 0.03 μg/kg (based on a signal/noise of 3:1) and 0.10 μg/kg, respectively.

For the evaluation of the method, samples of pig *Longissimus dorsi* muscle, pork ham, horse sausages and *L. vannamei* shrimps were analyzed to verify the absence of matrix interfering peaks in the retention time-window of the OTA peak: the comparison between a blank and a non-compliant sample demonstrating the absence around ± 2.5% of the OTA retention time of endogenous interference.

This study confirms that the method used is easy and time-saving, is reliable, accurate and can be applied at levels of OA contamination considerably lower than the maximum levels, which future European regulations are expected to allow for such food. Therefore, in agreement with Regulation (EC) No 882/2004 (Annex III) of the European Parliament and of the Council of 29 April 2004 [28] on official controls performed to ensure the verification of compliance with feed and food law, animal health and animal welfare rules, the optimized analytical method is characterized by accuracy, applicability, limit of detection, limit of determination, precision, repeatability, reproducibility, recovery, selectivity, sensitivity, linearity and measurement uncertainty.

The concentrations of OTA found in the tissues of the wild boar sampled are shown in Table 1. The highest levels of OTA were found in the kidneys of the 23 wild boar analyzed (0.1–3.9 μg/kg, average 1.1 ± 1.15 μg/kg). The levels found in the liver and urinary bladder were two-fold lower than in the kidneys (liver: 0.1–2 μg/kg, average 0.5 ± 0.54; urinary bladder: 0.1–2.6 μg/kg, average 0.6 ± 0.58). The lowest contents were found in muscle (0.1–1.3 μg/kg, average 0.3 ± 0.26). In 12 of the tissue samples examined in this study (seven kidney, two liver, two bladder and one muscle), the levels of OTA were higher than the guideline level (1 μg/kg) established by the Italian Ministry of Health Circular No 10 dated 9 June 1999. 

**Table 1:** Ochratoxin A concentration (μg/kg) in the kidney, liver, urinary bladder and muscle of wild boar.

Samples	Kidney	Liver	Urinary bladder	Muscle
1	1.9	0,9	0.3	0.5
2	1.4	1.0	1.0	0.3
3	0.3	0.2	0.9	0.3
4	1.1	0.3	0.2	0.2
5	0.3	0.2	0.2	0.1
6	3.2	1.8	2.6	1.3
7	0.4	0.2	0.4	0.3
8	2.2	0.2	0.2	0.2
9	0.3	0.2	0.3	0.1
10	0.7	0.1	0.6	0.4
11	0.4	0.1	0.8	0.3
12	0.2	0.9	0.2	0.1
13	0.9	0.2	0.3	0.2
14	0.1	0.9	0.2	0.3
15	1.0	0.3	0.8	0.2
16	3.9	0.7	0.3	0.2
17	0.3	0.1	0.3	0.1
18	0.3	0.1	0.2	0.1
19	0.8	0.2	0.3	0.1
20	3.8	2.0	1.7	0.5
21	0.4	0.2	0.3	0.1
22	0.8	0.2	0.6	0.4
23	0.3	0.1	0.1	0.1

In the 23 animals included in this study, gross and microscopic lesions were observed in the kidney, liver and urinary bladder. Gross kidney lesions were found in samples #1, #2, #6, #16 and #20 that had the highest OTA levels: 1.9 μg/kg, 1.4 μg/kg, 3.2 μg/kg, 3.9 μg/kg and 3.8 μg/kg, respectively. 

**Figure 1:**
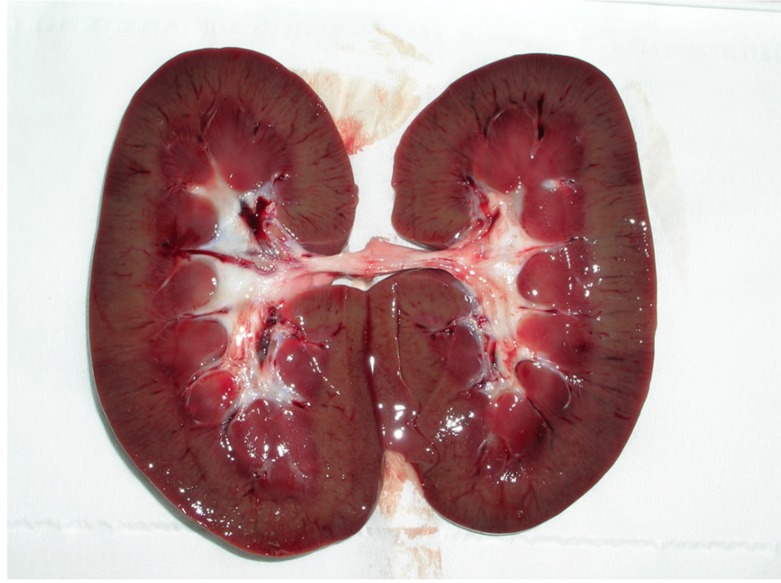
Porcine kidney: Enlargement of pelvis and cortical hyperemia.

The most obvious lesions were seen in animals #6 and #20, where the kidneys appeared pale, soft and had enlargement of the pelvis, cortical hyperemia and red streaks arranged radially, suggesting cortex infiltration (Figure 1) [15,29]. 

**Figure 2:**
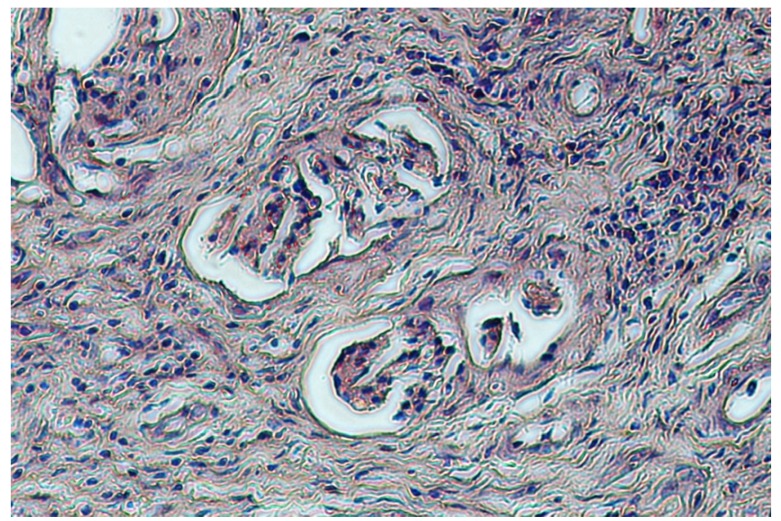
Porcine kidney: proliferation of Bowman’s capsule and nephron atrophy (H&E stain, 20×).

**Figure 3:**
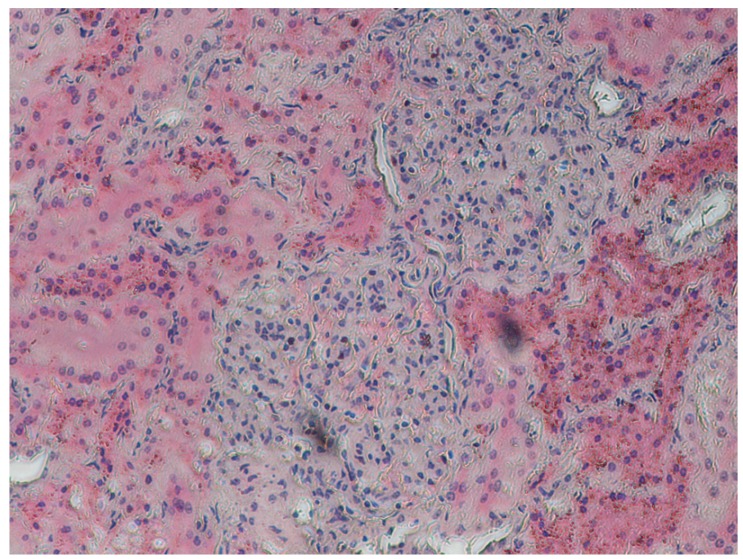
Porcine kidney: Compensatory functional hypertrophyof glomeruli and expanded tubules with oedema (H&E stain, 40×).

The kidney of animal #20 also showed a superficial fibrosis, suggesting chronic inflammation, which was confirmed by histopathological examination. Histology also revealed glomerular sclerosis and a proliferation of Bowman’s capsule that involved irreversible glomerular destruction and subsequent nephron atrophy (Figure 2). Connected to the fibrosis areas were compensatory functional hypertrophy of glomeruli and expanded tubules with oedema (Figure 3).

**Figure 4:**
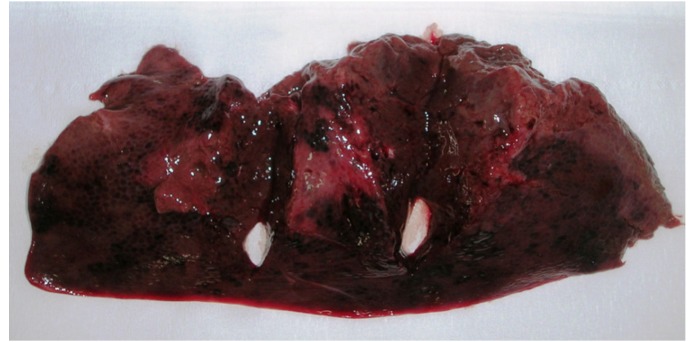
Porcine liver: Thinning of the margins of hepatic lobules, hyperemic areas and presence of dark/red alternated areas.

Gross lesions of the liver were seen in samples #6 and #20 that had the highest OTA levels: 1.8 μg/kg and 2 μg/kg, respectively. These samples showed evidence of hepatic lobular thinning at the margins, and the surface of the organ presented alternating dark-red areas (Figure 4).

**Figure 5:**
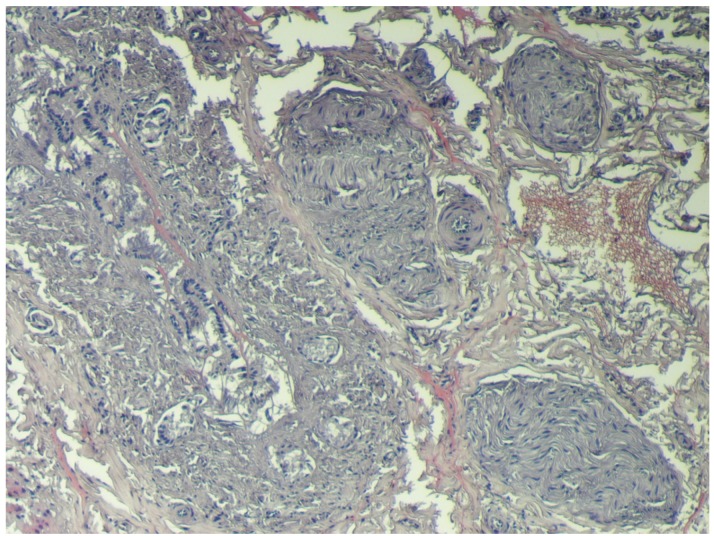
Porcine liver: Proliferation of the fibroblastic connective tissue around the bile duct and veins (H&E stain, 10×).

The histological sections showed a small number of hepatocytes with fatty infiltration. Additionally, groups of vacuolated hepatocytes and necrotic cells were found scattered throughout the liver parenchyma. There were considerable proliferations of the fibroblastic connective tissue around bile ducts and veins (Figure 5).

**Figure 6:**
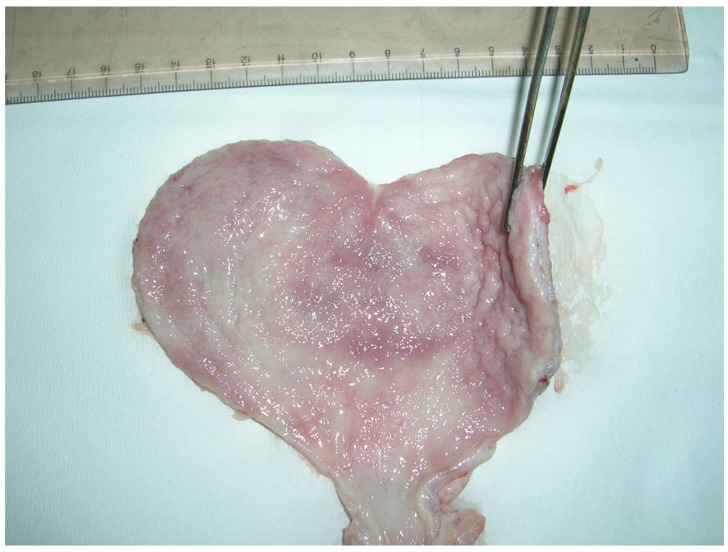
Porcine urinary bladder: Abundant mucus, hyperemia and hemorrhagic areas and thickening of the wall.

Gross and histopathological lesions of the urinary bladder were found in tissue samples #6 and #20 that had the highest OTA levels: 2.6 μg/kg and 1.7 μg/kg, respectively. Macroscopic observation showed: hyperemic mucosa and thickening of the wall; abundant mucus associated with hyperemia and hemorrhagic areas (Figure 6). 

**Figure 7:**
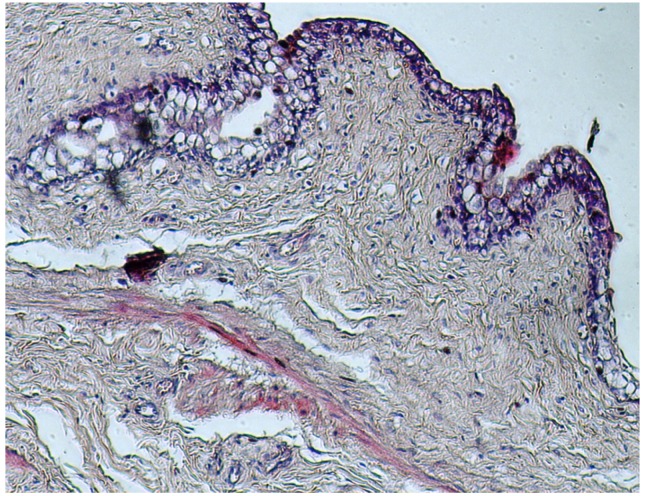
Urinary bladder: Proliferation of the fibroblastic connective tissue, disorders of transitional epithelial cells and presence of vacuolated cytoplasm (H&E stain, 40×).

Histological examination of the urinary bladder showed extensive proliferation of fibroblastic connective tissue, focal infiltration by inflammatory cells, degenerative changes in transitional epithelial cells and areas with vacuolated cytoplasm (Figure 7).

In all of the animals, OTA was detected in the kidneys, liver, urinary bladder and muscle and is in agreement with two recent studies on porcine nephropathy and OTA detection in tissues [25,30], with OTA concentrations evident, in the decreasing order of: kidney, urinary bladder, liver and muscle.

## 4. Discussion

On the basis of our findings, the histopathological alterations in the wild boar exposed to OTA, were more evident in the kidney and urinary bladder than in the liver. Similar histopathological changes were described in swine fed with OTA by Ceci *et al.* [25], but the levels in swine organs were six- and three-times higher than in the wild boar, respectively. Due to the lack information on wild boar nutrition, it is not possible to exclude the possibility that these histopathological alterations may represent a chronic state. 

Traditionally, in the Calabria region and in several other Italian regions (Umbria, Tuscany, Lazio and Basilicata), wild boar meats are used to produce niche products, especially coppa and salami. In agreement with the study of Monaci *et al*. [26], dried wild boar meat may contribute to overall OTA intake by carry-over effects into processed meats. This hypothesis was considered in Question N° EFSA-Q-2005-154 [20], which reported that it cannot be excluded that regular consumption of certain regional specialties, not yet considered in the assessment of exposure, contributes to human OTA exposure, especially in teenagers, whose relatively low body weight as compared to adults results in a higher exposure per kg body-weight.

Our data suggest that there is a need for new inspection and sanitary control measures associated with the consumption of these niche products to reduce the entry of OTA into the human diet. In order to ensure food safety, all of the steps in the food chain need to be controlled, which is probably not possible for these products. It would therefore be opportune to apply the precautionary principle until new epidemiological studies have been carried out regarding the quantification of OTA in wild boar tissues or meat products derived from these animals. In addition, as indicated by the guidelines of the European Community (Commission Regulation (EC) No 178/2002) [31], monitoring the quality of meat destined for transformation is a priority in order to decrease the possibility of toxin carry-over to humans.

## 5. Conclusions

The findings of our study demonstrate that histopathological alterations in the wild boar exposed to OTA are more evident in the kidney and urinary bladder rather than in the liver.

The presence of OTA residues and pathological lesions in tissues of OTA-exposed animals is a concern for human and animal health. 

Moreover, the present study confirms that contamination of meat products by OTA represents a potential emerging source of OTA for distinct segments of the Italian population, who are significant consumers of locally-produced wild boar specialties. 
